# Risk perceptions and changes in tobacco use in relation to Coronavirus Disease 2019 pandemic: A qualitative study on adolescent tobacco users in Hong Kong

**DOI:** 10.18332/tid/167479

**Published:** 2023-07-14

**Authors:** Tianqi Chen, Lijun Wang, Yee Tak Derek Cheung, Man Ping Wang, Tai Hing Lam, Sai Yin Ho

**Affiliations:** 1 School of Public Health, The University of Hong Kong, Hong Kong SAR, People’s Republic of China; 2 School of Nursing, The University of Hong Kong, Hong Kong SAR, People’s Republic of China

**Keywords:** tobacco use, adolescents, COVID-19, e-cigarettes, risk perception

## Abstract

**INTRODUCTION:**

Tobacco use is associated with an increased risk of Coronavirus Disease 2019 (COVID-19) infection, severe COVID-19 outcomes requiring intensive care, and mortality. We investigated the perceived risk of and changes in cigarette, e-cigarette (EC) and heated tobacco product (HTP) use in relation to COVID-19 in Hong Kong adolescent tobacco users.

**METHODS:**

We conducted semi-structured telephone interviews from January to April 2021 and in February 2022 on 40 adolescents (63% boys, Secondary school grades 2–6) who participated in our previous smoking surveys and were using cigarettes, ECs or HTPs before the first wave of the COVID-19 pandemic in January 2020.

**RESULTS:**

Adolescents generally perceived higher risks of contracting and having more severe COVID-19 from using cigarettes than ECs/HTPs, but they had limited knowledge of COVID-19 risks from EC/HTP use, particularly. Both increased and reduced consumption were found in tobacco, with EC use being the less affected product. Changes also included switching to ECs for convenience and lower cost and shifting from smoking cigarettes outside to mainly at home or in hidden areas. COVID-related policies, fear of infection, non-COVID-related health concerns, less social opportunities and pocket money, and limited access to tobacco products were barriers to tobacco use. In contrast, greater freedom at home versus school and negative emotions due to social distancing were facilitators. Family/peer influence had mixed impacts.

**CONCLUSIONS:**

Adolescent tobacco users perceived lower COVID risks associated with HTPs and ECs than cigarettes, and various changes in tobacco use were found amid the pandemic in Hong Kong. COVID-19 and related social changes may both facilitate or deter adolescent tobacco use.

## INTRODUCTION

Tobacco use is the leading cause of preventable deaths worldwide and is associated with increased risks of lung diseases, respiratory infections, and other communicable diseases^1^. COVID-19 poses additional risks to tobacco users, as they are considered more susceptible to exacerbated symptoms of COVID-19^2^. The impaired lungs and weakened immune systems render smokers more vulnerable to the COVID-19 virus^3^. Moreover, our prior study suggests that smokers are more likely to contract COVID-19 because the virus may spread through frequent hand-to-mouth contact^4^. The evidence on e-cigarette (EC) use, though relatively limited, also suggests an increased risk of infection due to hand-to-mouth action, sharing of devices and the transmission of the virus in EC aerosols, and more severe outcomes due to the weakened respiratory system^5^.

Tobacco users respond differently to COVID-19. The pandemic might have led to reduced tobacco use or increased quit attempts due to raised health concerns among the public^6-8^ or by hindering access to cigarettes and social opportunities for smoking through COVID-related restrictions (such as lockdowns and reduced social gatherings)^9^. Conversely, increased tobacco use during the pandemic has also been reported in some countries (France, Iceland, USA and Brazil), potentially due to adverse psychosocial outcomes like anxiety and depression^10-13^, the need to relieve boredom during lockdowns and the perception of smoking as a coping mechanism for stress^6^. The COVID-19 outbreak in Hong Kong experienced two peaks in 2020, with the first peak on 9 February and a second wave in March. Sparing a city-wide lockdown, the Hong Kong Special Administrative Region government implemented stringent measures to contain the spread of the virus, including quarantine, social distancing, and school suspensions. Our previous study on adult tobacco users in Hong Kong found an 11.5% net decrease in overall tobacco use (cigarettes, ECs, HTPs) after the first two waves of the COVID-19 outbreak^14^.

Compared with the general population, young people are more susceptible to the adverse effects of social isolation^15^ and experience greater mental health problems^16^. They may engage more in risk-taking behaviors during the COVID-19 pandemic, partly due to their different risk perceptions^17^. A longitudinal study in the US of young adults found that lower perceived smoking risk predicted the initiation of cigarette use^18^. In US adolescents (aged 13–17 years), the perception that ‘EC use would increase one’s risk for contracting COVID-19’ was inversely correlated with past 30-day EC use^19^. However, the risk perception and changes in different tobacco products in adolescents during COVID-19 have not been reported.

While several quantitative studies have investigated changes in tobacco use due to COVID-19 in adolescents^20,21^, these studies rely on predetermined survey questions. They might not capture the complexity and variability of adolescents’ experiences in this context. Herein, we investigated the harm perceptions towards different tobacco products (cigarettes, ECs, and HTPs) and the impact of COVID-19 on behavioral changes in tobacco use among Hong Kong adolescent tobacco users.

## METHODS

**Figure 1:**
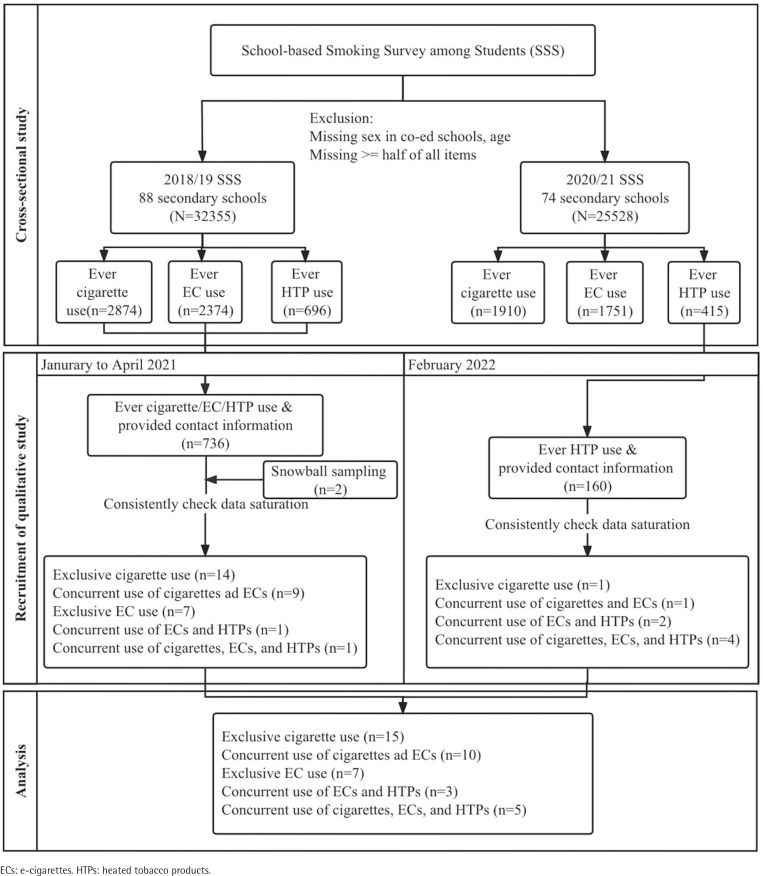
Flow diagram of the recruitment process of the qualitative study

Adolescent tobacco users of cigarettes, ECs and HTPs were selected from the participants of the School-based Smoking Survey among Students in 2018–2019 (conducted before COVID-19) and 2020–2021 (conducted since the fourth wave of the COVID-19 outbreak in Hong Kong). The territory-wide crosssectional survey was conducted every two years among Hong Kong secondary school students (grades 2–6, typically aged 13–17 years). Students reported their tobacco use behaviors in detail and provided contact numbers for potential follow-up studies. An overview of the recruitment process is shown in Figure 1.

Eligible students were current users of any of the above three products before the outbreak of COVID-19 (December 2019 to January 2020) based on the question, ‘Before the outbreak of COVID-19, in December 2019, which of the following tobacco products were you using? (cigarettes, ECs or HTPs)’. From January to April 2021, we phoned all 736 tobacco users identified from the 2018–2019 round survey. Student tobacco users were also recruited by referral of existing participants. Data saturation was obtained from the first 13 cigarette users and 15 EC users. We identified more students who reported HTP use (n=160) with contact information in the 2020–2021 round survey and recruited eight current HTP users. Current cigarette users and EC users were also included in the analysis among those who reported ever HTP use in February 2022.

Telephone interviews were conducted using an interview guide (Supplementary file Part 1) covering two topics: 1) the perceived association of tobacco use with susceptibility to contracting COVID-19 and vulnerability to serious COVID-19 consequences by different tobacco products; and 2) the perceived impacts of the pandemic on tobacco use behaviors and changes in tobacco use since the outbreak of COVID-19. The experiences of tobacco use in both the early phase of the COVID-19 outbreak (February–March 2020) and later phases (July–August 2020 for Wave 3, November–December 2020 for Wave 4) were asked and collected, including changes in tobacco consumption, places of use, type of tobacco products and the underlying reasons. Interviews were conducted by three trained research staff. All interviewees provided verbal informed consent and were given incentives with a HK$100 (US$12.8) cash transfer or supermarket coupon after the interview. Ethics approval (No. UW 21-011) was granted by the Institutional Review Board of the University of Hong Kong/Hospital Authority Hong Kong West Cluster.

Analysis was led by two researchers, WL and CT. All interviews were audio-recorded and transcribed verbatim by four research assistants. The transcripts of each transcriber were randomly selected for accuracy checking. Data were prepared for analysis using NVivo qualitative software (version 12.0) (QSR International Pty. Ltd., 2012, Doncaster, Australia). Inductive coding and narrative analysis methods were adopted^22^ to construct the thematic framework and potential subthemes. Then, the transcripts and coded data were re-examined to build individual narratives and case histories. All transcripts were double-coded, with regular meetings held to review data by two researchers and a senior researcher. Disagreements were reconciled through discussion.

## RESULTS

### Participants and characteristics

Table 1 shows that of 40 participants, 63% (n=25) were boys, and 46% (n=18) were in Secondary school grade 6. Exclusive cigarette use was most common (n=15), followed by concurrent use of cigarettes and ECs (n=10), exclusive ECs use (n= 7), concurrent use of all three tobacco products (n=5), and concurrent use of ECs and HTPs (n=3). No one used HTPs exclusively. In total, 30 of them used cigarettes, 25 used ECs and 8 used HTPs.

**Table 1:** Demographic and tobacco-related characteristics of participants (N=40)

*Characteristics*	*n*	%*
**Sex**		
Boy	25	63
Girl	15	38
**Age (years),** mean ± SD	14.5 ± 2.1^a^	
**Grade**		
Secondary 2	4	10
Secondary 3	9	23
Secondary 4	2	5
Secondary 5	7	18
Secondary 6	18	45
**Tobacco use status before the outbreak of COVID-19**		
Exclusive cigarette use	15	38
Concurrent use of cigarettes and ECs	10	25
Exclusive EC use	7	18
Concurrent use of ECs and HTPs	3	8
Concurrent use of cigarettes, ECs, and HTPs	5	13

ECs: e-cigarettes. HTPs: heated tobacco products.

a Age was estimated from Student Enrolment Statistics in 2020–2021 from Education Bureau, The Government of the Hong Kong SAR.

* Due to rounding, the percentages may not add up to 100%.

**Figure 2:**
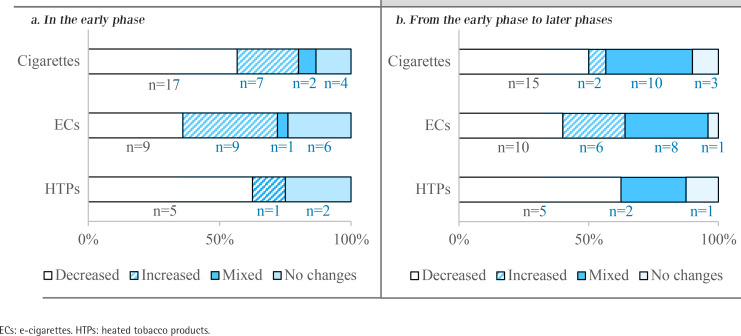
Changes in specific tobacco use in the early phase (Feb–Mar 2020) and later phases (July–Aug 2020, Nov–Dec 2020) of the COVID-19 pandemic

Figure 2 shows the major changes in tobacco use during the COVID-19 pandemic. Compared with the pre-pandemic phase, more cigarette users decreased their cigarette consumption than increased in both the early (17 vs 7) and later phases (15 vs 2) of COVID-19. An equal number of EC users decreased and increased their use in the early phase (9 vs 9), while more decreased than increased (10 vs 6) in later phases; 5 of 8 HTP users decreased their use in the early and later phases.

Additionally, 7 participants reported using new tobacco products during the pandemic: 3 cigarette users used ECs, 1 EC user used cigarettes, one dual user of cigarette and EC used HTPs, and two dual users of ECs and HTPs used waterpipe. Further details of changes in the tobacco use behaviors of all participants are shown in Supplementary file Table 1.

### Perceived association between tobacco use and COVID-19

Participants held varying opinions on the relationship between tobacco use and COVID-19. Most participants considered tobacco users to be at a higher risk of contracting and would experience severe symptoms of COVID-19. According to the participants, this perception was influenced by several factors, including the potential for exposure to the virus when smoking without a mask, poor hand hygiene, worsened health outcomes and weakened immune systems associated with tobacco use.

Some participants, however, believed that tobacco users were at no greater risk of contracting COVID-19 or would not experience severe symptoms than non-tobacco users. They attributed the risk of contracting COVID-19 to personal hygiene practices and luck, and the severity of symptoms to general health. Additionally, one participant perceived a lower risk of infection or severity of COVID-19 associated with tobacco use due to the ‘protective function’ of substances in cigarettes.

Participants held conflicting views regarding the harmfulness of different tobacco products, with some perceiving ECs and HTPs as less harmful than traditional cigarettes, and EC/HTP users were at similar or lower risk of contracting COVID-19 or would experience less severe symptoms if infected.

Over half of the participants had limited knowledge (n=23) about the risks associated with EC/HTP use and COVID-19 and had limited access to reliable resources of information (n=22) on the association between tobacco use and COVID-19.

The most common sources of information of participants were ‘personal knowledge/experience’, ‘social media/online information’, ‘official news/TV’, and ‘friends’ experience’. Only a few participants (n=3) had ever learned about the association between tobacco use and COVID-19 from ‘schools or classrooms’. Further details of participants’ perceptions regarding the association between tobacco use and COVID-19 are shown in Supplementary file Table 2.

**Table 2:** Participants’ perceived impacts of COVID-19 and changes in tobacco use behaviors

*Impacts*	*Representative quotes of changes in tobacco use behaviors*
**Barriers**	
**COVID-19 policies**	*‘I usually find a small alley or a place with few people [if there is a need to smoke outside]. It is not as easy to be caught by the police.’* (No. 30, Boy, S6, cigarettes exclusively)
	*‘I am scared that if I did not wear a mask and were found by the Food and Environmental Hygiene Department or the police, I would be fined HK$5000.’* (No. 16, Girl, S6, cigarettes and ECs)
	*‘The epidemic helps me quit smoking because wearing a mask is so troublesome. I am too lazy to buy cigarettes, and there are fewer channels to buy cigarettes. Moreover, I rarely go out and have few chances to hang out with friends. In fact, there are many factors around to help you quit.’* (No. 12, Boy, S6, cigarettes and ECs)
**Fear of COVID-19 infection**	*‘I smoke less because I had the idea to do so in the past and because I learned that COVID-19 is more common in smokers.’* (No. 21, Boy, S2, ECs exclusively)
**Non-COVID-related health concerns**	*‘I smoked less due to health concerns. When I started to wear masks, I felt breathless and experienced shortness of breath.’* (No. 19, Boy, S3, cigarettes exclusively)
	*‘I had been thinking about quitting smoking even before the pandemic. Regardless of the pandemic, I have worried about my lung function getting worse.’* (No. 29, Girl, S5, cigarettes, ECs and HTPs)
**Fewer social opportunities**	*‘Smoking is probably largely socially motivated; that is, more social activities may make me smoke more. Since the epidemic outbreak, I rarely go out, participate in fewer social activities, and smoke fewer cigarettes.’* (No. 10, Girl, S4, cigarettes exclusively)
**Financial issues**	*‘Because when I have no money, I smoke less and more slowly. I will only buy cigarettes after I have worked and get paid.’* (No. 30, Boy, S6, cigarettes exclusively)
**Limited access to tobacco products**	*‘I go out less frequently (due to COVID-19) and do not bother going out just to buy cigarettes. Since that, I have since used to smoking fewer cigarettes.’* (No. 18, Boy, S6, cigarettes exclusively)
	*‘I could not find channels for purchasing ECs because nearby shops have closed due to the pandemic. So I changed to using cigarettes because you can buy them at any convenience stores or newspaper stands.’* (No. 2, Boy, S2, cigarettes and ECs)
**Facilitators**	
**Transition to online learning**	*‘Smoking is not allowed at school. But after I took online classes at home, no one restrained me. Thus, I smoke more than before.’* (No. 7, Boy, S3, cigarettes exclusively)
**Negative emotions**	*‘The need to take exams despite not understanding the teachers fully on Zoom classes stressed me out. So I smoked more.’* (No. 13, Boy, S3, ECs only)
	*‘I can’t go to many places to relieve myself when I’m having problems because of the pandemic. So I turn to smoking to alleviate the situation.’* (No. 15, Girl, S6, cigarettes and ECs)
**Mixed impacts**	
**Family/friend influence**	*‘My family dislikes my smoking. I always feel anxious and apprehensive when I go back home [after smoking], and then I feel that smoking is so meaningless.’* (No. 1, Boy, S3, ECs and HTPs)
	*‘I was invited to my friend’s home and tried waterpipes several times during the pandemic.’* (No. 11, Boy, S5, ECs and HTPs)

ECs: e-cigarettes. HTPs: heated tobacco products.

### Perceived COVID-19 impacts and changes in tobacco use behaviors: barriers and facilitators

According to participants’ responses, we found nine perceived impacts of the pandemic on tobacco use behaviors (Table 2). Of these, six were barriers to tobacco use, including COVID-19 policies, fear of COVID-19 infection, non-COVID-related health concerns, fewer social opportunities, financial issues, and limited access to tobacco products. Two factors were facilitators of tobacco use: the transition to online learning and negative emotions. The family/peer influence on individuals’ tobacco use behaviors during the epidemic had both positive and negative effects. Changes were found in tobacco consumption, smoking places and the type of tobacco products used. Additional information on COVID-19 impacts and changes in tobacco use is available in Supplementary file Table 3.

*Barriers*

The COVID-19 policies were a common factor that influenced adolescent tobacco use. Participants had shifted tobacco use from outside to mainly at home or inconspicuous public places as masking was mandatory by law with no exemption for smoking.

Many participants reduced or quit tobacco use in response to COVID-19 policies, which restricted smoking outside through measures such as mandatory masking regulations and high fines (HK$5000). The prolonged period spent at home due to social distancing restrictions and school closures also contributed to this change, particularly since many participants lived with their families and were unable to smoke at home due to disapproval from family members.

Another barrier to adolescent tobacco use is the fear of COVID-19 infection, which has reduced or quit tobacco use in some participants. They reported concerns about the heightened risk of infection associated with smoking due to the need to remove masks in public places and inhale deeply.

In addition to the direct impact of COVID-19, the pandemic has heightened the concerns of adolescents about their health and led to decreased tobacco use in many. Some adolescents also experienced health problems or perceived their situation as worsening during the pandemic, which further prompted them to decrease tobacco use.

Many participants reduced their cigarette use due to the limited social interaction opportunities caused by social isolation. To reduce smoking, several participants actively reduced socializing with smoking friends, and one even ‘cut off contact with smoking friends’.

Several participants decreased tobacco use or switched to other tobacco products due to the difficulty of access to tobacco products and financial concerns amid the pandemic. A few participants reported no change in tobacco consumption as they still had access to tobacco products online without age limit restrictions:

*‘I bought ECs online, such as from IG shops, because you don't need to provide an ID card for age verification.’* (No. 35, Girl, S2, ECs and HTPs)

*Facilitators*

Participants reported that the transition to online learning led to increased tobacco use due to a lack of supervision and boredom.

Several participants expressed that the transition to online learning led them to smoke more because they had difficulty focusing on schoolwork and could not follow a structured schedule or prepare for exams in the same way as they would in a physical classroom.

Negative emotions, such as stress, anxiety and loneliness during the pandemic, was a factor in increased tobacco use. Participants expressed that smoking helped them to relieve stress and anxiety, and to pass the time during periods of social isolation.

*Mixed impacts and other changes*

The impact of family/peer influence during the pandemic was mixed. Some participants reported reducing tobacco use due to discouragement from their family or peer group. Others, however, reported that their peer group either introduced them to new tobacco products or activities provided tobacco products to them, leading to trying new tobacco products.

EC use was less affected than cigarette/HTP use during the pandemic. Some participants found ECs more convenient to use indoors or outside while wearing a mask as it produces less smoke and odor. Even though the long time spent at home restricted the cigarette use of some participants, the reduced cigarette use was compensated by increased EC use in some participants.

Some participants reported increased tobacco consumption in later phases of COVID-19 due to the ‘anti-pandemic fatigue’. As they grew accustomed to the ongoing pandemic and COVID-19 restrictions, they resumed smoking despite the health risks.

## DISCUSSION

This is a novel qualitative study investigating adolescents’ harm perception towards tobacco use and changes in tobacco use in the pandemic. The results showed that adolescents had limited knowledge of the risk of COVID-19 from tobacco use, particularly from EC/HTP use. Their risk perception of tobacco use in relation to COVID-19 was based on personal experiences or observations of friends rather than from WHO, government departments or other authoritative or credible sources. More adolescents had decreased than increased tobacco consumption while others showed a mixed pattern of changes with decreases and increases. EC use was the least affected. A few tobacco users had initiated the use of other products, including ECs/HTPs and waterpipe. Some switched from cigarettes to ECs or HTPs or both, and from smoking outside to smoking mainly at home or in hidden places outside. We found more barriers than facilitators of tobacco use, but family/peer influence had mixed impacts.

Our results are consistent with a qualitative study that reported much confusion in US young adults on the association between tobacco use and COVID-19, especially for EC use compared with cigarette use^23^. Results on the association between COVID-19 and tobacco use, particularly ECs and HTPs during the early stage of the pandemic were scarce in the literature, and in reports and messages in mass and social media. The limited knowledge and sources of reliable information, and hence such perception and confusion, are not unexpected in our adolescents, and in young adults and adults elsewhere. Such results could reflect the failure of timely and effective communication to the whole population in many countries to use the facts about the known harms of tobacco use to promote tobacco control amid the pandemic. As more results on the associations between COVID-19 and various tobacco products are emerging, the opportunities to use such results for tobacco control should not be missed again.

We found both increases and decreases in tobacco use among adolescents, but more adolescents had decreased tobacco use than increased. This finding is consistent with cross-sectional studies in Turkish^24^ and Indonesian adolescents^25^, which reported greater decreases than increases in adolescent tobacco use amid the COVID-19 pandemic. But these studies did not investigate differences in changes in different tobacco products. Our qualitative study addressed this gap in adolescents and found that EC use was less affected than cigarette/HTP use amid the pandemic. Some participants even reported increased consumption of ECs or switching from cigarettes/HTPs to ECs when smoking at home. This aligns with a study in US young people that reported an increase in EC use due to changes in daily environment that made EC use a more feasible option^26^. Adolescents would feel that they could use them more freely than cigarettes in places where conventional cigarettes or HTPs would be more easily detected, such as in their homes or at school^27^. Notably, we found easy access to cigarettes or ECs in adolescents, despite that sales to minors are prohibited. In contrast to a previous study in US youth and young adults that reported barriers to EC access due to COVID-19 policies^28^, only one adolescent in our study reported difficulty in buying EC pods. In Hong Kong, even though the manufacture, import, advertising, distribution, and sale of ECs and HTPs have been banned since 30 April 2022, the use and non-commercial possession of such products are not prohibited. Whether the new regulations have been successful in minimizing adolescent use of such products is uncertain. These findings are warning and suggest an urgent need for strong enforcement of the regulations and monitoring of the impacts on adolescent tobacco use in Hong Kong.

We found more barriers than facilitators to tobacco use in adolescents amid COVID-19. COVID-19 policies, especially masking mandates and social gathering restrictions, have served as a deterrent, resulting in reduced tobacco consumption and smoking outside in our study. Some adolescents changed from smoking openly to secluded places such as small alleys or turning to ‘fast smoking’ to avoid the heavy fines and reduce the risk of infection from unmasking and smoking. The deterring effect was great, as this fine (HK$5000, US$1=HK$7.8) is much greater than that of HK$1500 for smoking in places where smoking is prohibited. These behaviors were also observed in US young adults^23^, US adults^29^ and Hong Kong adults^30^. After Hong Kong lifted the mandatory mask-wearing requirement on 1 March 2023^31^, concerns about an increase in smoking prevalence after opening up have been raised by the government, tobacco control advocates and in the mass media.

Our study found that spending more time at home due to social gathering restrictions and fewer social opportunities decreased adolescent tobacco use, which is consistent with previous studies on US college students^32^ and adolescents^21^. Importantly, our findings found that ‘anti-pandemic fatigue’ during later phases of the COVID-19 pandemic led some participants to resume increased tobacco use, resulting in mixed changes in adolescent smoking behavior. These findings suggest that the pandemic might have only temporarily deterred tobacco use in adolescents. As we approach the end of the COVID-19 pandemic, further studies are necessary to assess the long-term impact of the pandemic on tobacco use in adolescents.

The other barriers to adolescent tobacco use in our study included financial problems, limited access to tobacco products, fear of COVID-19 infection and non-COVID-related health concerns. These factors discouraged tobacco use in many adolescents but not all. In our study, financial problems generally led to decreased smoking frequency and consumption. These findings support that raising the tobacco tax is an effective strategy for deterring young people from using tobacco products, especially for those not financially well off. Although the pandemic did not directly affect the availability of cigarettes in retail stores, as Hong Kong had no lockdown, many adolescents reported going out less often due to fear of infection. While such fear was not the most commonly reported influential factor, young smokers generally perceived themselves to be at a lower risk of COVID-19 infection and its health consequences compared to adult smokers^33^. Whether emphasis on the risks of COVID-19 and increasing fear would motivate more adolescents to quit smoking is uncertain.

Our study found greater freedom at home and negative emotions as major facilitators of tobacco use among adolescents. In Hong Kong, as in other regions with school closures, the shift to online learning during the pandemic might have contributed to increased EC use. Students had more time to stay at home and less supervised time than in schools where smoking is not permitted, especially when they are alone at home. Furthermore, negative emotions associated with the pandemic, such as boredom or academic pressure, could have further exacerbated these facilitators of smoking. A survey study on Canadian adolescents showed that tobacco use was an avoidant stress-coping strategy in the early phase of the pandemic^34,35^. The sudden shift to remote learning presented great challenges for adolescents, with some having difficulty transitioning to virtual learning amid the pandemic^36^. Finally, the results of more barriers than facilitators of tobacco use corroborated the results of more adolescents decreased than increased tobacco use. As the pandemic is nearing its end and the barriers associated with COVID-19 are diminishing, our results support the worry of increased tobacco use after the relaxation of all control measures and the opening up in Hong Kong and elsewhere. Further studies and monitoring on the effects of opening up on tobacco and nicotine product use in adolescents (as well as in adults) are needed to inform policies.

### Limitations

Our study has some limitations. First, tobacco use status was self-reported without validation by biochemical tests. Second, the number of HTP users was small as their use was less common than that of ECs and cigarettes. Third, whether our results could be generalized to other places with different COVID-19 control measures and smoking prevalence is uncertain. Additionally, the qualitative design of our study did not allow for statistical inference, so the findings should be interpreted with caution. Lastly, it is important to note that the results might not be applicable to different age groups, as our focus was on adolescents in Hong Kong.

## CONCLUSIONS

Adolescents had limited sources of reliable information on COVID-19 risks from tobacco use, especially from ECs and HTPs. While more adolescents had decreased than increased tobacco consumption during the pandemic, EC use was the least affected. Tobacco use had shifted from outside to mainly at home or hidden areas. COVID-19 and related social changes may both facilitate or deter adolescent tobacco use. The insights gained from our study can inform ongoing research to reduce tobacco use and future policy development to respond to crises such as COVID-19. Even though the pandemic is fading, closely monitoring the long-term impact of tobacco use remains necessary.

## Data Availability

The data supporting this research are available from the corresponding author at syho@hku.hk, on reasonable request.

## References

[1] World Health Organization (2022). Coronavirus disease (COVID-19): Tobacco.

[2] Gülsen A, Yigitbas BA, Uslu B, Drömann D, Kilinc O (2020). The effect of smoking on COVID-19 symptom severity: systematic review and meta-analysis. Pulm Med.

[3] Vardavas CI, Nikitara K (2020). COVID-19 and smoking: a systematic review of the evidence. Tob Induc Dis.

[4] Sun Y, Lam TH, Cheung YTD (2021). First report on smoking and infection control behaviours at outdoor hotspots during the COVID-19 pandemic: an unobtrusive observational study. Int J Environ Res Public Health.

[5] Besaratinia A, Tommasi S (2021). The consequential impact of JUUL on youth vaping and the landscape of tobacco products: the state of play in the COVID-19 era. Prev Med Rep.

[6] Almeda N, Gómez-Gómez I (2022). The impact of the COVID-19 pandemic on smoking consumption: a systematic review of longitudinal studies. Front Psychiatry.

[7] Rigotti NA, Chang Y, Regan S (2021). Cigarette smoking and risk perceptions during the COVID-19 pandemic reported by recently hospitalized participants in a smoking cessation trial. J Gen Intern Med.

[8] Ghadban YA, Zgheib N, Romani M, Akl IB, Nasr R (2022). Impact of the COVID-19 pandemic on smoking behavior and beliefs among the American University of Beirut community. Tob Prev Cessat.

[9] Filby S, van der Zee K, van Walbeek C (2022). The temporary ban on tobacco sales in South Africa: lessons for endgame strategies. Tob Control.

[10] Thorisdottir IE, Asgeirsdottir BB, Kristjansson AL (2021). Depressive symptoms, mental wellbeing, and substance use among adolescents before and during the COVID-19 pandemic in Iceland: a longitudinal, population-based study. Lancet Psychiatry.

[11] Pelham WE 3rd, Tapert SF, Gonzalez MR (2021). Early adolescent substance use before and during the COVID-19 pandemic: a longitudinal survey in the ABCD study cohort. J Adolesc Health.

[12] Rolland B, Haesebaert F, Zante E, Benyamina A, Haesebaert J, Franck N (2020). Global changes and factors of increase in caloric/salty food intake, screen use, and substance use during the early COVID-19 containment phase in the general population in France: survey study. JMIR Public Health Surveill.

[13] Malta DC, Szwarcwald CL, Barros MBdA (2020). The COVID-19 pandemic and changes in adult Brazilian lifestyles: a cross-sectional study, 2020. Epidemiol Serv Saude.

[14] Sun Y, Wang MP, Cheung YTD (2022). Changes in tobacco use at the early stage of the COVID-19 pandemic: results of four cross-sectional surveys in Hong Kong. Tob Induc Dis.

[15] Beam CR, Kim AJ (2020). Psychological sequelae of social isolation and loneliness might be a larger problem in young adults than older adults. Psychol Trauma.

[16] Varma P, Junge M, Meaklim H, Jackson ML (2021). Younger people are more vulnerable to stress, anxiety and depression during COVID-19 pandemic: A global cross-sectional survey. Prog Neuropsychopharmacol Biol Psychiatry.

[17] Wolfe K, Sirota M, Clarke ADF (2021). Age differences in COVID-19 risk-taking, and the relationship with risk attitude and numerical ability. R Soc Open Sci.

[18] Bennett B, Romm KF, Berg CJ (2022). Changes in cigarette and e-cigarette use among US young adults from before to during the COVID-19 pandemic: news exposure and risk perceptions as potential predictors. Tob Prev Cessat.

[19] Cai X, Zhao X, Rossheim ME, Xue H (2021). Vaping and COVID-19 risk: perceived link and its correlates among at-risk adolescents. Prev Med Rep.

[20] Clendennen SL, Case KR, Sumbe A, Mantey DS, Mason EJ, Harrell MB (2021). Stress, dependence, and COVID-19-related changes in past 30-day marijuana, electronic cigarette, and cigarette use among youth and young adults. Tob Use Insights.

[21] Wharton MK, Islam S, Abadi MH, Pokhrel P, Lipperman-Kreda S (2023). COVID-19 restrictions and adolescent cigarette and e-cigarette use in California. Am J Prev Med.

[22] Braun V, Clarke V (2006). Using thematic analysis in psychology. Qualitative Research in Psychology.

[23] Cassidy RN, Bello MS, Denlinger-Apte R (2023). The impact of the COVID-19 pandemic on a sample of US young adults who smoke cigarettes: a qualitative analysis. Addict Behav.

[24] Taş D, Üneri ÖŞ (2023). COVID-19 quarantine effects on smoking behavior and mental health of smoking adolescents. Eurasian J Med.

[25] Sen LT, Siste K, Hanafi E (2021). Insights into adolescents’ substance use in a low–Middle-Income Country during the COVID-19 pandemic. Front Psychiatry.

[26] Clausen M, Romm KF, Berg CJ (2022). Exploring young adults’ e-cigarette use behavior during COVID-19. Tob Prev Cessat.

[27] Ramamurthi D, Chau C, Jackler RK (journal). JUUL and other stealth vaporisers: hiding the habit from parents and teachers. Tob Control.

[28] Gaiha SM, Lempert LK, Halpern-Felsher B (2020). Underage youth and young adult e-cigarette use and access before and during the Coronavirus Disease 2019 pandemic. JAMA Netw Open.

[29] Maloney SF, Combs M, Scholtes RL (2021). Impacts of COVID-19 on cigarette use, smoking behaviors, and tobacco purchasing behaviors. Drug Alcohol Depend.

[30] Zhang X, Sun Y, Cheung DYT (2022). Changes in tobacco use in the early phase of Coronavirus Disease 2019 pandemic in Hong Kong: a qualitative study. Nicotine Tob Res.

[31] The Government of the Hong Kong Special Administrative Region (2023). Government lifts all mandatory mask-wearing requirements.

[32] Sokolovsky AW, Hertel AW, Micalizzi L, White HR, Hayes KL, Jackson KM (2021). Preliminary impact of the COVID-19 pandemic on smoking and vaping in college students. Addict Behav.

[33] Lam KK, Ho KY, Wu CS, Tong MN, Tang LN, Mak YW (2022). Exploring factors contributing to the smoking behaviour among Hong Kong Chinese young smokers during COVID-19 pandemic: a qualitative study. Int J Environ Res Public Health.

[34] Schnitzer K, Jones S, Kelley JHK, Tindle HA, Rigotti NA, Kruse GR (2021). A qualitative study of the impact of COVID-19 on smoking behavior for participants in a post-hospitalization smoking cessation trial. Int J Environ Res Public Health.

[35] Riazi NA, Battista K, Duncan MJ (2023). Stronger together: coping behaviours and mental health changes of Canadian adolescents in early phases of the COVID-19 pandemic. BMC Public Health.

[36] Cockerham D, Lin L, Ndolo S, Schwartz M (2021). Voices of the students: adolescent well-being and social interactions during the emergent shift to online learning environments. Educ Inf Technol.

