# Corrigendum: Risk perceptions and changes in tobacco use in relation to Coronavirus Disease 2019 pandemic: A qualitative study on adolescent tobacco users in Hong Kong

**DOI:** 10.18332/tid/176535

**Published:** 2024-02-12

**Authors:** Tianqi Chen, Lijun Wang, Yee Tak Derek Cheung, Man Ping Wang, Tai Hing Lam, Sai Yin Ho

**Affiliations:** 1 School of Public Health, The University of Hong Kong, Hong Kong SAR, People’s Republic of China; 2 School of Nursing, The University of Hong Kong, Hong Kong SAR, People’s Republic of China

**Keywords:** tobacco use, adolescents, COVID-19, e-cigarettes, risk perception

Corrigendum on:

Risk perceptions and changes in tobacco use in relation to Coronavirus Disease 2019 pandemic: A qualitative study on adolescent tobacco users in Hong Kong

By Tianqi Chen, Lijun Wang, Yee Tak Derek Cheung, Man Ping Wang, Tai Hing Lam, Sai Yin Ho

Tobacco Induced Diseases, Volume 21, Issue July, Pages 1-11,

Publish date: 14 July 2023

DOI: https://doi.org/10.18332/tid/167479

In the originally published version of the article, in the Methods section of the Abstract, “65%” is now replaced by “63%”. In the 4th page, 1st paragraph of the left column, the phrase “Ethics approval was granted by the Institutional Review Board of the University of Hong Kong/ Hospital Authority Hong Kong West Cluster”, has been changed into “Ethics approval (No. UW21-011) was granted by the Institutional Review Board of the University of Hong Kong/ Hospital Authority Hong Kong West Cluster.”

In the 4th page, 4th paragraph of the left column, the phrase “Fewer EC users decreased their use than increased in the early phase (9 vs 10), while more decreased than increased (11 vs 6) in later phases;” is now replaced by “An equal number of EC users decreased and increased their use in the early phase (9 vs 9), while more decreased than increased (10 vs 6) in later phases;”

Finally, in Figure 2, 5th page, have been added the categories “a. In the early phase” and “b. From the early phase to later phases”. The mentioned changes have been corrected also online.

