# Supplementary material for: Risk perceptions and changes in tobacco use in relation to Coronavirus Disease 2019 pandemic: A qualitative study on adolescent tobacco users in Hong Kong
Source: Tob Induc Dis. 2023 Jul 14;21:92. doi: 10.18332/tid/167479 (PMC10347963; doi:10.18332/tid/167479)
Supplement: Supplementary file 1 [file TID-21-92-s1.pdf]

Supplementary Appendix 1: Interview guide

Screening question:

<table>
<tr><td colspan="3">In the period before the outbreak of COVID-19, from December 2019 to January 2020, were you using any of the following tobacco products?</td></tr>
<tr><td>a. Cigarettes</td><td>a. Yes</td><td>b. No</td></tr>
<tr><td>b. Electronic cigarettes</td><td>a. Yes</td><td>b. No</td></tr>
<tr><td>a. Heated tobacco products</td><td>c. Yes</td><td>d. No</td></tr>
<tr><td colspan="3">-- Choosing <u>b</u> for <u>all the three products</u> —> I'm sorry, but this interview is mainly focused on long-term smokers. Thank you very much for your answers. Goodbye.
--Choosing <u>a OR b</u> for <u>any of the three products</u> —> Okay, I see that you meet the requirements for this interview. Let's officially begin the interview now. Don't be nervous, just answer based on your own situation, and say whatever comes to mind</td></tr>
</table>

Interview guide:

Topic 1: Perception towards COVID-19 and tobacco use

1.1. Association between tobacco use and COVID-19 infection.

1.1.1. What do you think of the probability of contracting COVID-19 in cigarette users and never tobacco users, higher, lower or similar? Why?

1.1.2. What do you think of the probability of contracting COVID-19 in EC users and never tobacco users, higher, lower or similar? Why?

1.1.3. What do you think of the probability of contracting COVID-19 in HTP users and never tobacco users, higher, lower or similar? Why?

1.2. Association between tobacco use and severity of COVID-19

1.2.1. Compared to people who do not smoke, if cigarette users contract COVID-19, will their symptoms be milder, more severe, or the same? Why?"

1.2.2. Compared to people who do not smoke, if EC users contract COVID-19, will their symptoms be milder, more severe, or the same? Why?"

1.2.3. Compared to people who do not smoke, if HTP users contract COVID-19, will their symptoms be milder, more severe, or the same? Why?"

1.3. Sources of information

1.3.1. Where did you get the information on the associations between COVID-19 and tobacco use?

Topic 2: Experience in tobacco use amid the pandemic.

Next, we would like to understand the impact of your tobacco use on your experience with COVID-19

2.1 Changes in tobacco use behaviours

2.1.1 Compared to the pre-pandemic period, when the outbreak started in Hong Kong (about February/March 2020), how have you changed your consumption of cigarettes/ECs/HTPs

2.1.2 Except for the aforementioned tobacco products you used, did you start using other tobacco products during the pandemic? And the frequency?

2.1.3 Have the following factors impacted your smoking behaviour, such as the amount of tobacco you consume and where you smoke (at home or outdoors)? (The factors included health concerns, social-distancing restrictions, mandatory masking regulations, work from home/ study at home, financial issues, access to tobacco products, and other reasons)

2.1.4 Were your tobacco use behaviours in the later phase of the pandemic (July-August 2020 for Wave 3, November-December 2020 for Wave 4) consistent with those of the early phase

(February-March 2020)? Type of tobacco use, consumption, access to tobacco products, and places of use

2.2 Reasons for changes in tobacco use behaviours

2.2.1 **Reasons for decreased or want to decrease.**

2.2.1.1 What had prompted you to decrease? Please list.

2.2.1.2 What were some barriers, if any, that you encountered when you decreased tobacco use?

2.2.1.3 How did you overcome the barrier(s)?

2.2.1.4 Are there any barriers that you have not overcome? Please describe.

2.2.2 Reasons for increased

2.2.2.1 What had prompted you to increase? Please list

2.2.3 Reasons for no change

2.2.3.1 As you said before that you want to decrease smoking since learning of COVID-19, but you did not change smoking behaviours during COVID-19. So what were some barriers that you encountered? Or what are the difficulties of decreasing tobacco use for you?

2.2.4 If changed in terms of types of tobacco products, amount of consumption, accessibility or smoking location, ask why.

3 CLOSING

3.1 The in-depth interview is about to end. Would you like to add anything that you think is important but not covered in the interview?

3.2 Thank you very much for your participation. We have gained a lot of valuable insights from your sharing and experience. Thank you!

(Stop recording)

Supplementary Appendix 2: Supplementary Table 1

Supplementary Table 1. Summary of changes in tobacco use in the early phase and later phases of the COVID-19 pandemic (N=40)

Changes in tobacco use	**Exclusive users**		**Concurrent users**			Total
	Cigarettes (n=15)	ECs (n=7)	Cigarettes & ECs (n=10)	ECs &HTPs (n=3)	Cigarette, ECs & HTPs (n=5)	n (%)
**The early phase of COVID-19**						
Decreased						
Cigarettes	9	-	5	-	3	17
ECs	-	2	6	0	1	9
HTPs	-	-	-	1	4	5
Increased						
Cigarettes	4	-	3	-	0	7
ECs	-	2	4	1	2	9
HTPs	-	-	-	1	0	1
Mixed						
Cigarettes	0	-	1	-	1	2
ECs	-	0	0	1	0	1
HTPs	-	-	-	0	0	0
No change						
Cigarettes	2	-	1	-	1	4
ECs	-	3	0	1	2	6
HTPs	-	-	-	1	1	2
**From the early phase to later phases of COVID-19**						
Decreased						
Cigarettes	7	-	5	-	3	15
ECs	-	2	6	0	2	10
HTPs	-	-	-	1	4	5
Increased						
Cigarettes	1	-	1	-	0	2
ECs	-	3	2	0	1	6
HTPs	-	-	-	0	0	0
Mixed						
Cigarettes	5	-	4	-	1	10
ECs	-	2	2	3	1	8
HTPs	-	-	-	2	0	2
No change						
Cigarettes	2	-	0	-	1	3
ECs	-	0	0	0	1	1
HTPs	-	-	-	0	1	1
**First experimental use since the COVID-19**						
Cigarettes	-	1	-	0	0	1

ECs	3	-	-	-	0	3
HTPs	0	0	1	-	0	1
Waterpipe	0	0	0	2	-	2

Abbreviations: ECs, e-cigarettes; HTPs, heated tobacco products.

Supplementary Appendix 3. Supplementary Table 2

Supplementary Table 2. Summary of participants' perceptions on the association between tobacco use and COVID-19			
	Cigarette	EC	HTP
**Perceived susceptibility to contracting COVID-19 in tobacco users versus non-users**			
**More susceptible**	*(n=23)*<br>*They have to take off their mask in public places to smoke, and smokers have to inhale deeply. The risk of contracting COVID-19 is undoubtedly higher than for non-smokers. (No. 23, Girl, S6, cigarettes and ECs)*<br><br>*I think the risk is higher for cigarette users. Because if you smoke, you have worse immunity and are more likely to be infected by COVID-19. (No.8, Boy, S6, cigarettes exclusively)*	*(n=22)*<br>*People who smoke e-cigarettes have a higher risk of infection because sometimes they share e-cigarettes and have the saliva contact. It is possible, as I have seen that before. (No. 13, Boy, S3, EC exclusively)*<br><br>*Because I think e-cigarettes have the same effect on the lungs. Lung function and health will be damaged. (No. 9, Boy, S6, EC exclusively)*	*(n=14)*<br>*I think people who take off their masks and smoke HTPs on the street are at higher risk than non-smokers. As they are mostly older, and they usually smoke HTP and talk together. (No. 7, Boy, S3, cigarettes exclusively)*<br><br>*Because they are all cigarettes, HTPs will also damage lung function. (No. 28, Girl, S6, ECs and HTPs)*
**Less susceptible**	*(n=3)*<br>*Tobacco products contain nicotine, nicotine and the virus are all harmful substances. I heard that it may be possible to kill the new coronavirus. (No. 2, Boy, S6, cigarettes and ECs)*	*(n=1)*<br>*Lower risk, because most of my friends who use HTPs are not infected even if they removed their masks and smoked outside, but my friends who did not use tobacco were infected. (No.*	*(n=3)*<br>*Similar to the quotes in cigarettes and ECs*

		11, Boy, S5, ECs and HTPs)	
**Similar susceptibility**	*(n=12)*<br>*It seems smoking is not related to COVID-19. I believe that people who smoke cigarettes will not be infected if they practise good hygiene. (No. 32, Girl, S5, cigarettes and ECs)*<br><br>*I think COVID-19 infection is more related to the body's immunity. (No. 25, Boy, S3, cigarettes exclusively)*<br><br>*Most cases of COVID-19 are elderly, and few are young. COVID-19 is age-related; it has nothing to do with smoking. (No. 37, Girl, S5, cigarettes, ECs and HTPs)*	*(n=12)*<br>*ECs do not contain nicotine and do not cause much damage, so the risk is about the same as non-smokers. (No, 34, Girl, S2, cigarettes and ECs)*<br><br>*About half of my friends who smoke and half of my friends who don't smoke were infected with COVID-19, so I don't think smoking is related to the infection. (No. 40, Girl, S6, cigarettes, ECs and HTPs)*<br><br>*Although they (cigarettes &ECs) are all harmful to the human body, EC users seldom smoke outside, most of them smoke at home. Usually, you just take a puff or two and then wear the mask and stop smoking. So I think the risk is almost the same as non-smokers. (No. 39, Girl, S5, cigarettes exclusively)*	*(n=7)*<br>*COVID-19 is more related to personal hygiene, I think the risk of infection is not increased in HTP users if they have good personal hygiene habits. (No. 24, Boy, S6, cigarettes, ECs and HTPs)*

**Not clear**	*(n=2)* *There should be no difference (between people who smoke cigarettes/ECs/HTP and non-smokers) ... I don't know. I can't even think about it. (No. 20, Boy, S6, cigarettes exclusively)*	*(n=5)* *I just don't know why. (No. 37, Girl, cigarettes, ECs and HTPs)*	*(n=16)* *I am not clear what this product is, as well as its association with COVID-19. (No. 1, Boy, S3, cigarettes and ECs)*
**Perceived vulnerability to serious COVID-19 consequences in tobacco users versus non-users**			
**More vulnerable**	*(n=26)* *Because some chemicals in cigarettes have already damaged the lungs of users, the symptoms should be more severe if they are infected. (No. 12, Boy, S5, cigarettes, ECs and HTPs)*<br><br>*Early symptoms of COVID-19 are commonly present in chronic smokers, so it may take them longer to confirm infection, hence more severe. (No. 22, Girl, S6, ECs exclusively)*	*(n=19)* *In fact, both ECs and traditional cigarettes can damage lung function. They would suffer more severe symptoms if they were infected with COVID-19 and may have more sequelae. (No. 13, Boy, S3, ECs exclusively)*	*(n=15)* *The chemical composition of HTPs and cigarettes is almost the same. Thus HTPs should be as harmful as cigarettes. (No. 23, Girl, S6, cigarettes, ECs and HTPs)*
**Less vulnerable**	*NA*	*(n=4)* *Chemicals in ECs could prevent the invasion of bacteria and viruses and might also*	*(n=2)* *Symptoms are milder. EC/HTP users have more tar or other chemicals in their lungs, so*

		protect the lung from severe symptoms. (No. 2, Boy, S6, cigarettes and ECs)	*bacteria are less likely to invade. (No. 11, Boy, S5, ECs and HTPs)*
**Similar vulnerability**	*(n=10)* *I think it depends more on the general health of individuals than the smoking status (No. 4, Girl, S6, cigarettes exclusively)*	*(n=10)* *ECs are not as harmful as cigarettes. Although ECs also contain harmful substances such as nicotine, the nicotine content is much lower than in cigarettes. Therefore, the lung function of EC users will not be seriously damaged, so even if they become infected with COVID-19, the symptoms will not be particularly exaggerated. (No. 14, Boy, S6, cigarettes exclusively)*	*(n=9)* *Regardless of smoking habits, once people are infected, the symptoms are the same, and it is equally hard. (No. 37, Girl, S3, cigarettes, ECs and HTPs)*
**Not clear**	*(n=4)* *I think there are many factors (related to the severity), so it is hard to say whether the symptoms of tobacco users would be milder or more severe if they were infected. (No. 17, Boy, S6, cigarettes exclusively)*<br><br>*I can't imagine there is any association between smoking and*	*(n=7)* *Because I do not know much about COVID-19, and even if I knew that smoking harms my health, I could not feel it because I smoke ECs and do regular exercise, so I have a very good cardiorespiratory function. Hence, I just don't know. (No. 33, Girl, S5, ECs exclusively)*	*(n=14)* *Few people smoke HTPs around me, so I don't know. (No. 27, Boy, S6, cigarettes exclusively)*

	COVID-19. (No. 7, S3, Boy, cigarettes exclusively)		

Abbreviations: ECs, e-cigarettes; HTPs, heated tobacco products.

Supplementary Appendix 4: Supplementary Table 3

Supplementary Table 3. Summary of participants' perceived impacts of COVID-19 and changes in tobacco use behaviours	
**Impacts**	**Tobacco use behaviours**
**COVID-19 policies**	-Change the smoking location to home<br>*I mostly smoked at home (after the dine-in ban), for I never go out and only order takeout. (No. 14, Boy, S6, cigarettes exclusively)*
	-Change the smoking location to less crowded places<br>*I do not smoke in crowded streets or in districts with many confirmed cases. (No. 10, Girls, S4, cigarettes exclusively)*<br>*I will not light a cigarette in the streets, take off my mask and smoke while walking. I usually find a small alley or a place with few people (if there is a need to smoke outside). It is not as easy to be caught by the police. (No. 30, Boy, S6, cigarettes exclusively)*
	-Decreased cigarette use<br>· Social distancing restriction<br>*In the past, I smoked in streets every day, but my family did not allow me to go outside due to the Social Distancing Restriction after COVID-19, so I cannot smoke now. (No. 24, Boy, S6, cigarettes, ECs and HTPs)*<br>· Dine-in bans<br>*After dining in was banned, I went out less and had fewer chances to dine with my friends. Thus, I smoked less. (No. 17, Boy, S6, cigarettes exclusively)*<br>· Masking regulation<br>*Yes, the masking regulation (has an impact on my cigarette use). I am scared that if I did not wear a mask and were found by the Food and Environmental Hygiene Department or the police, I would be fined HK$5000. (No. 16, Girl, S6, cigarettes and ECs)*<br>· School closure<br>*I could not smoke cigarettes both at home and at school. Before COVID-19, I always smoked with my friends after school before the pandemic. Now I study from home*

	and have little chance to smoke. (No. 10, Girl, S4, cigarettes exclusively)
	-Quit cigarette use<br>*I started to quit smoking after the outbreak of the fourth wave of the pandemic. I seldom go out and spend most of my time at home. I am quite a clean freak and don't want to smoke at home. (No. 23, Girl, S6, cigarettes and ECs)*<br>*The epidemic helps me quit smoking because wearing a mask is so troublesome. I am too lazy to buy cigarettes, and there are fewer channels to buy cigarettes. Moreover, I rarely go out and have few chances to hang out with friends. In fact, there are many factors around to help you quit. (No. 12, Boy, S6, cigarettes and ECs)*
	-Unaffected EC or HTP use<br>*My vaping was not affected by COVID-19, as I could just put the pod inside my mask and smoke outside. ECs are smokeless and odourless, so I can vape at home, and my family wouldn't know that I smoke. (No. 35, Girl, S2, ECs and HTPs)*
	-Decreased cigarette but increased EC use<br>*I could not smoke cigarettes at home, so I smoked ECs instead. (No. 8, Boy, S6, cigarettes exclusively)*
**Fear of COVID-19 infection**	-Change the smoking location to home<br>*Because of the fear of contracting COVID-19, I cut down on smoking cigarettes outside, so I usually return home to smoke. (No. 15, Girl, S6, cigarettes and ECs)*
	-Decreased cigarette use<br>*I smoke less because I had the idea to do so in the past and because I learned that COVID-19 is more common in smokers. (No. 21, Boy, S2, ECs exclusively)*
	-Quit cigarette use<br>*I quit smoking after seeing many studies pointing to an increased risk of COVID-19 infection and harm to my body because I am so worried about my health. (No. 19, Boy, S3, cigarettes exclusively)*
	- Decreased EC use<br>*Because I am afraid (of the infection). No matter whether you smoke outside or inside, you are afraid of bacteria and viruses. (No. 13, Boy, S5, ECs exclusively)*
	-Decreased and then increased cigarette use in later phases<br>*We were all afraid when the pandemic unexpectedly struck us. We were unsure of the potential consequences of*

	the disease, which was extremely terrifying. But over time, we become accustomed to it. (No. 31, Boy, S4, cigarettes and ECs)
**Non-COVID-related health concerns**	-Decreased cigarette use<br>*I smoked less due to health concerns. When I started to wear masks, I felt breathless and experienced shortness of breath. (No. 19, Boy, S3, cigarettes exclusively)*<br>*Actually, I had been thinking about quitting smoking even before the pandemic. Regardless of the pandemic, I have worried about my lung function getting worse. (No. 29, Girl, S5, cigarettes, ECs and HTPs)*
	-Quit cigarette use<br>*In the past, I smoked two packs of cigarettes per day, and then I noticed that my health condition became worse, and my skin became terrible. Girls care a lot about their skin. It has a huge impact on my general status. Therefore, I realised that I must quit it. (No. 15, Girl, S2, cigarettes and ECs)*
	-Decreased EC use<br>*One of my friends smoked ECs for a long time. Once, I accompanied him to the hospital for a health check-up, finding his lungs quite white. I had never known that ECs do such damage to our health and so I started to smoke less. (No. 31, Boy, S4, cigarettes and ECs)*
**Fewer social opportunities**	-Decreased cigarette use<br>*Smoking is probably largely socially motivated; that is, more social activities may make me smoke more. Since the epidemic outbreak, I rarely go out, participate in fewer social activities and smoke fewer cigarettes. (No. 10, Girl, S4, cigarettes exclusively)*
	-Decreased EC use<br>*I smoke mainly when socialising with a bunch of friends; it makes me chill. But I am not addicted to tobacco and do not need to buy an EC pod myself. Now I spend less time with my friends and thus do not smoke. (No. 26, Boy, S3, ECs exclusively)*
**Financial issues**	-Decreased cigarette use<br>*Because when I have no money, I smoke less and more slowly. I will only buy cigarettes after I have worked and get paid. (No.30, Boy, S6, cigarettes exclusively)*
	-Decreased EC use<br>*Of course, the EC device costs too much. Take JUUL as an example; a pod is too expensive that I hesitate to buy,*

	so I smoke less. (No. 41, Boy, S2, cigarettes, ECs and HTPs)<br>-Decreased cigarette but increased EC use<br>*Yes, the financial issue (influenced my EC use). An EC pod can be smoked for many days, so it feels not expensive. An EC pod costs HK$30, and it can be smoked for a much longer time than cigarettes. (No. 32, Girl, S5, cigarettes and ECs)*
**Limited access to tobacco products**	-Decreased cigarette use<br>*I go out less frequently (due to COVID-19) and do not bother going out just to buy cigarettes. Since that, I have been used to smoking fewer cigarettes. (No. 18, Boy, S6, cigarettes exclusively)*<br>-Quit cigarette use<br>*Because I rarely go out and don't want to go out on purpose to buy cigarettes, I gradually quit smoking. (No. 34, Boy, S2, cigarettes and ECs)*<br>-Decreased EC use<br>*A friend of mine was an EC agent before, so she/he directly gave me pods for free, but later she/he did not give me (pods) due to supply issues, and I didn't bother to buy them, so I just smoked less. (No. 16, Girl, S2, cigarettes and ECs)*<br>-Change EC to cigarette use<br>*I could not find channels for purchasing ECs because nearby shops have closed due to the pandemic. So I changed to using cigarettes because you can buy them at any convenience stores or newspaper stands. (No. 2, Boy, S2, cigarettes and ECs)*
**Transition to online learning**	-Increased cigarette use<br>*Smoking is not allowed at school. But after I took online classes at home, no one restrained me. Thus, I smoke more than before. (No. 7, Boy, S3, cigarettes exclusively)*<br>-Increased EC use<br>*My smoking increased because I had more time than before, especially smoking ECs; sometimes, I smoked ECs during virtual classes. (No. 41, Boy, S2, cigarettes, ECs and HTPs)*<br>*The need to take exams despite not understanding the teachers fully on Zoom classes stressed me out. So I smoked more.s (No. 13, Boy, S3, ECs only)*<br>*Because I can't smoke cigarettes during the online lesson, I can only smoke them at night after classes and*

	smoke more ECs at home due to the online study. (No. 38, Boy, S3, cigarettes and ECs)
**Negative emotions**	-Increased cigarette use<br>*When preparing for the exam, I was under pressure and then smoked (cigarettes) a lot. (No. 16, Girl, S2, cigarettes and ECs)*<br>*I spent a lot of time in the house during the epidemic, and I was too bored in the house. So I smoked more. (No. 32, Girl, S5, cigarettes and ECs)*<br>*I can't go to many places to relieve myself when I'm having problems because of the pandemic. So I turn to smoke to alleviate the situation. (No. 15, Girl, S6, cigarettes and ECs)*
	-Increased EC use<br>*When I am in a bad mood, I need to smoke (ECs) more to make me feel better. (No. 11, Boy, S5, ECs and HTPs)*<br>*Due to staying at home, I smoke ECs much more than before (the pandemic). Staying long at home is boring, so I used ECs like eating snacks. (No. 31, Boy, S4, cigarettes and ECs)*<br>*I enjoy going out but could only stay at home for so many days (during the pandemic). It made me unhappy, so I smoked when I was lonely and depressed. (No. 28, Girl, S6, ECs and HTPs)*
**Family/friend influence**	-Decreased cigarette use<br>*My family dislikes my smoking. I always feel anxious and apprehensive when I go back home (after smoking), and then I feel that smoking is so meaningless. (No. 1, Boy, S3, ECs and HTPs)*<br>*I smoke less because it's not worth causing trouble and making my family angry. It's both expensive and troublesome for me to buy and smoke. (No. 26, Boy, S6, EC exclusively)*
	-Quit cigarette & EC use<br>*Actually, many of my friends don't smoke, and friends at school mind smoking behaviour; that is, if you smoke, you might be an "MK boy" or a bad student. I want to build good relationships with my classmates because I care about what other people think, so I quit smoking cigarettes and ECs. (No. 31, Boy, S4, cigarettes and ECs)*
	-Obstacle to cessation

	I had attempted to quit before but failed because I would have the urge to smoke when my friends around me were smoking". (No. 38, Boy, S3, cigarettes and ECs)
	-First try of new tobacco products<br>*I was invited to my friend's home and tried waterpipes several times during the pandemic. (No. 11, Boy, S5, ECs and HTPs)*<br>*My friend ever let me smoke his e-cigarettes, but I felt that it was different from cigarettes, so I gave it up. (No. 17, Boy, S6, cigarettes exclusively)*

Abbreviations: ECs, e-cigarettes; HTPs, heated tobacco products.

© 2023 Chen T. et al.
